# Ixodid ticks (Acari: Ixodidae) collected from African savanna elephants (*Loxodonta africana*) and African forest elephants (*Loxodonta cyclotis*)

**DOI:** 10.4102/ojvr.v86i1.1781

**Published:** 2019-11-06

**Authors:** Edward Kariuki, Hellen Kutima, Michael Kock, Ivan G. Horak, Roaland Jooste, Luis Neves

**Affiliations:** 1 Department of Veterinary Services, Kenya Wildlife Service, Nairobi, Kenya; 2 School of Biomedical Sciences, Jomo Kenyatta University of Agriculture and Technology, Nairobi, Kenya; 3 Department of Zoology, Jomo Kenyatta University of Agriculture and Technology, Nairobi, Kenya; 4 International Wildlife Veterinary Services, Greyton, South Africa; 5 Department of Veterinary Tropical Diseases, Faculty of Veterinary Science, University of Pretoria, Pretoria, South Africa; 6 Animal Health Division, Bayer (Pty) Ltd, Isando, Johannesburg, South Africa

**Keywords:** African savanna elephants, African forest elephants, ixodid ticks, Republic of Congo, Kenya

## Abstract

Eight ixodid tick species were collected from 173 African savanna elephants (*Loxodonta africana*) in Kenya, northern Mozambique and Zimbabwe, and two species were collected from six African forest elephants (*Loxodonta cyclotis*) in the Republic of Congo. A new host record is reported for *Amblyomma eburneum*. A list of ticks collected from elephants in various African countries, and stored in the United States National Tick Collection, is supplied as well as an annotated checklist of the 27 ixodid tick species that have been collected from African elephants. The geographic distributions and alternative hosts of the various tick species collected from elephants are briefly discussed.

## Introduction

Despite the African savanna elephants’ iconic status, no surveys dedicated to the ticks of elephants as such have been conducted in Africa. Hoogstraal (1956) recorded collections of *Amblyomma tholloni* from four elephants in the Equatoria province, Sudan, and Theiler (1962) reported collections of eight *Amblyomma* spp. (two are doubtful records) from elephants in sub-Saharan Africa. Among these, *A. tholloni* was present in 18 countries from Sierra Leone and Sudan in the north to South Africa in the south. Theiler also reported collections of *Dermacentor circumguttatus* from elephants in 10 countries in West, Central and East Africa, from Sierra Leone in the west to Tanzania in the east and northern Mozambique in the south. Furthermore, she recorded the collection of 10 *Rhipicephalus* spp. from elephants, among which were three of the four ornate *Rhipicephalus* spp.: *Rhipicephalus humeralis, Rhipicephalus maculatus* and *Rhipicephalus pulchellus*. Unfortunately, Theiler did not record the number of elephants infested with each species. Yeoman and Walker (1967) reported collections from 19 elephants in Tanzania along with collections made from numerous other animals, including domestic livestock. The elephants were infested with four tick species and 16 animals were infested with *A. tholloni*, three with *D. circumguttatus* and four with *Rhipicephalus maculatus*. In Kenya, Walker (1974) recorded eight tick species in collections made from 78 elephants. Forty-four animals were infested with *A. tholloni*, 28 with *R. humeralis*, 12 with *R. maculatus* and nine with *Rhipicephalus praetextatus* (then referred to as *Rhipicephalus simus*). In addition to *A. tholloni* and *R. maculatus*, Baker and Keep (1970) included *Rhipicephalus appendiculatus, Rhipicephalus muehlensi* and *R. simus* in a checklist of the ticks found on elephants and other large wildlife species in the north-eastern reserves of KwaZulu-Natal, South Africa. *Amblyomma tholloni* was the only species in collections made from 29 elephants in Zimbabwe (Norval 1983). Including the above-mentioned *Rhipicephalus* species, Walker, Keirans and Horak (2000) list 15 *Rhipicephalus* species that have been collected from elephants, among which is the fourth ornate *Rhipicephalus* species, *R. dux*. In a review of the ticks of the Central African Republic, Uilenberg, Estrada-Peña and Thal (2013) recorded a number of historic as well as recent collections of *Amblyomma astrion, Amblyomma cohaerens, A. tholloni, Amblyomma variegatum, D. circumguttatus* and *Rhipicephalus longus* from elephants. In Namibia, 8 of 45 elephants examined in the Etosha National Park harboured only *Hyalomma truncatum*, while no ticks were collected from the remaining elephants (Turner et al. 2017). In South Africa, Horak et al. (2018) recorded 32 collections from elephants, of which one animal was infested with a solitary *Amblyomma hebraeum* female, 23 with *A. tholloni* and eight with *R. maculatus*.

Here, we report opportunistic collections made since 1988 from African savanna elephants in Kenya, northern Mozambique and Zimbabwe and from African forest elephants in the Republic of Congo. We also include a list of tick species collected from elephants and deposited in the United States National Tick Collection (USNTC) as well as an annotated checklist of the tick species collected to date from African elephants.

## Methods

The management or translocation of elephants usually necessitates that they are restrained by sedation. Between 1988 and 2019, ticks were collected opportunistically from 173 sedated African savanna elephants and six African forest elephants. The animals were examined visually and ticks removed both manually and by using forceps. The ticks from each elephant were placed in individually labelled vials containing 70% ethanol and transported to the laboratory, where they were identified under a stereoscopic microscope using conventional keys and descriptions. No total collections of ticks were made from any of the elephants because of the size of the animal, the shortage of workforce, difficult operating conditions and the usually short period between sedation and resuscitation. The countries of origin and the species and number of ticks collected have been summarised in tabular format.

### Ethical considerations

The Kenyatta University Ethics Review Committee (KU-ERC) reviewed and approved the study protocol. The National Commission for Science, Technology and Innovation (NACOSTI) and the Kenya Wildlife Service (KWS) authorised the field study in Kenya.

## Results

The number of elephants examined in Kenya, Mozambique, Zimbabwe and the Democratic Republic of Congo, and the species and number of ticks collected are summarised in Table 1.

**TABLE 1:** Species and number of ticks collected from savanna and forest elephants.

Country or tick species	Number infested	Number of ticks collected	
		Male	Female
**Kenya (*n* = 128)**			
*Amblyomma eburneum*	1	9	4
*Amblyomma tholloni*	113	894	375
*Rhipicephalus appendiculatus*	3	3	0
*Rhipicephalus compositus*	1	4	1
*Rhipicephalus humeralis*	18	353	121
*Rhipicephalus maculatus*	7	30	17
*Rhipicephalus praetextatus*	11	12	8
*Rhipicephalus pulchellus*	7	77	43
**Mozambique (*n* = 36)**			
*Amblyomma tholloni*	36	147	61
*Rhipicephalus maculatus*	8	5	4
**Zimbabwe (*n* = 9)**			
*Amblyomma tholloni*	9	66	24
**Democratic Republic of Congo (*n* = 6†)**			
*Amblyomma tholloni*	6	27	31
*Dermacentor circumguttatus*	5	10	10

*n* = number of elephants sampled.

† , forest elephants.

A total of 128 elephants were examined at 61 localities across Kenya and 1964 ticks were collected (Table 1). The adults of eight tick species were identified in the collections. Two species belonged to the genus *Amblyomma* and six to the genus *Rhipicephalus. Amblyomma tholloni* was the most prevalent species and was collected from 88.3% of the elephants. *Amblyomma eburneum* is the first record of this species for African elephants. Three ornate species of *Rhipicephalus* are present in Kenya: *R. humeralis, R. maculatus* and *R. pulchellus* (Walker et al. 2000), and all were collected from the elephants. Ticks were also collected from a sick elephant in the Shimba Hills National Reserve, Kenya. It was infested with large numbers of ticks, and more than 3000 *R. maculatus,* as well as some *A. tholloni*, were collected from the posterior surface of a single ear.

*Amblyomma tholloni* was collected from all 36 elephants sampled in the Reserva Nacional do Niassa, Niassa Province, Mozambique, and *R. maculatus* was present in eight of these animals (Table 1). The nine animals sampled in Zimbabwe were infested only with *A. tholloni.* Six forest elephants examined in the Odzala-Kokoua National Park, Republic of Congo, were all infested with *A. tholloni*, while *D. circumguttatus* was collected from five animals (Table 1).

The species present in historic collections of ticks from African elephants between 1905 and 1980 and deposited in the USNTC are listed in Table 2.

**TABLE 2:** Ticks collected from African elephants (1905–1980) and deposited in the United States National Tick Collection.

Country or tick species	Number of collections	Number of ticks collected	
		Male	Female
**Sierra Leone**			
*Amblyomma tholloni*	1	16	14
*Dermacentor circumguttatus*	1	2	1
**Ghana**			
*Dermacentor circumguttatus*	1	10	6
**Nigeria**			
*Amblyomma tholloni*	1	2	1
**Cameroon**			
*Dermacentor circumguttatus*	1	2	2
**Republic of Congo**			
*Dermacentor circumguttatus*	1	1	1
**Democratic Republic of Congo**			
*Amblyomma tholloni*	1	0	2
**Angola**			
*Amblyomma tholloni*	2	10	2
**Somalia**			
*Amblyomma tholloni*	1	1	0
*Rhipicephalus praetextatus*	1	1	1
*Rhipicephalus pravus*	1	1	0
**Sudan**			
*Amblyomma tholloni*	5	12	7
*Rhipicephalus praetextatus*	7	36	49
**Kenya**			
*Amblyomma tholloni*	6	100	11
*Rhipicephalus humeralis*	2	6	6
*Rhipicephalus maculatus*	8	286	343
**Uganda**			
*Amblyomma cohaerens*	1	3	2
*Amblyomma tholloni*	16	76	62
*Dermacentor circumguttatus*	11	35	13
*Rhipicephalus praetextatus*	2	7	5
**Tanzania**			
*Rhipicephalus maculatus*	1	10	10
**Malawi**			
*Amblyomma tholloni*	1	2	1
**Mozambique**			
*Amblyomma tholloni*	2	2	2
**Zimbabwe**			
*Amblyomma tholloni*	3	5	7

Ticks from elephants in 14 African countries were present in the USNTC. *Amblyomma tholloni* was collected from elephants in 10 countries, *D. circumguttatus* in five, *R. praetextatus* (as *R. simus*) in three and *R. maculatus* in two. *Rhipicephalus humeralis* was collected from two elephants in Kenya, *Amblyomma cohaerens* from a single animal in Uganda and *R. pravus* from a single animal in Somalia.

An annotated checklist of tick species recorded on African elephants is presented in Table 3.

**TABLE 3:** Annotated checklist of the tick species collected from African elephants.

No	Tick	References
1	*Amblyomma astrion*	Theiler (1962); Uilenberg et al. (2013)
2	*Amblyomma cohaerens*	Theiler (1962); Matthysse and Colbo (1987)
3	*Amblyomma eburneum*	Kariuki et al. (present article)
4	*Amblyomma gemma*	Theiler (1962)
5	*Amblyomma hebraeum*	Horak et al. (2018)
6	*Amblyomma sparsum*	Theiler (1962); Walker (1974)
7	*Amblyomma tholloni*	Hoogstraal (1956); Aeschlimann (1967); Yeoman and Walker (1967); Baker and Keep (1970); Walker (1974); Uilenberg et al. (2013); Kariuki et al. (present article)
8	*Amblyomma variegatum*	Theiler (1962); Uilenberg et al. (2013)
9	*Dermacentor circumguttatus*	Theiler (1962); Aeschlimann (1967); Yeoman and Walker (1967); Uilenberg et al. (2013); Kariuki et al. (present article)
10	*Dermacentor rhinocerinus*	Theiler (1962)
11	*Hyalomma truncatum*	Turner et al. (2017); Horak et al. (2018)
12	*Rhipicephalus appendiculatus*	Theiler (1962); Baker and Keep (1970); Walker et al. (2000); Kariuki et al. (present article)
13	*Rhipicephalus compositus*	Theiler (1962); Kariuki et al. (present article)
14	*Rhipicephalus dux*	Walker et al. (2000)
15	*Rhipicephalus evertsi evertsi*	Walker (1974); Walker et al. (2000)
16	*Rhipicephalus humeralis*	Theiler (1962); Walker (1974); Walker et al. (2000); Kariuki et al. (present article)
17	*Rhipicephalus kochi*	Walker et al. (2000)
18	*Rhipicephalus longus*	Theiler (1962); Walker et al. (2000); Uilenberg et al. (2013)
19	*Rhipicephalus maculatus*	Theiler (1962); Yeoman and Walker (1967); Baker and Keep (1970); Walker (1974); Walker et al. (2000); Kariuki et al. (present article)
20	*Rhipicephalus muehlensi*	Theiler (1962); Baker and Keep (1970); Walker et al. (2000)
21	*Rhipicephalus planus*	Walker et al. (2000)
22	*Rhipicephalus praetextatus*	Walker et al. (2000); Fyumagwa et al. (2007); Kariuki et al. (present article)
23	*Rhipicephalus pravus*	Theiler (1962); Walker (1974); Walker et al. (2000)
24	*Rhipicephalus pulchellus*	Theiler (1962); Walker (1974); Walker et al. (2000); Kariuki et al. (present article)
25	*Rhipicephalus senegalensis*	Theiler (1962); Walker et al. (2000)
26	*Rhipicephalus simus*	Theiler (1962); Baker and Keep (1970); Walker et al. (2000)
27	*Rhipicephalus ziemanni*	Walker et al. (2000)

A total of 27 ixodid tick species have been collected from African elephants, and eight *Amblyomma* species, 16 *Rhipicephalus* species as well as *D. circumguttatus, D. rhinocerinus* and *Hyalomma truncatum* have been reported.

## Discussion

The discussion will focus on the tick species collected in the current survey.

### *Amblyomma* species

*Amblyomma eburneum* is a relatively rare species. Yeoman and Walker (1967) recorded it in the Eastern Province, Tanzania, while according to Walker (1974), ‘The few records of *A. eburneum* in Kenya are widely scattered’, where it has been found mainly in the centre and south of the country. Here, we report the first record on the African elephant. Collections have, however, been made from buffaloes in Tanzania (Yeoman & Walker 1967), cattle, black rhinoceroses, buffaloes, an oryx and a warthog in Kenya (Walker 1974), and buffaloes in Mozambique (Dias 1993).

*Amblyomma tholloni* was present in the majority of collections made from elephants in Kenya and Tanzania as well as in all the collections made from these animals in Zimbabwe (Norval 1983; Walker 1974; Yeoman & Walker 1967). The collection of *A. tholloni* from elephants in Kenya, the Republic of Congo, northern Mozambique and Zimbabwe in the present study confirms its widespread geographical distribution on African elephants. In Zimbabwe, however, Norval (1983) recorded *A. tholloni* on 22 of 24 hippopotamuses from which ticks had been collected. One of the two hippopotamuses we examined in Kenya was infested with *A. tholloni*. Ticks were collected from the inside of the lips, the nostrils, an ear and the skinfolds of the neck of this hippopotamus.

MacKenzie and Norval (1980) demonstrated that *A. tholloni* could transmit *Ehrlichia ruminatium*, the causative organism of heartwater in cattle, sheep and goats in the laboratory. They also noted that cattle, sheep and goats at a research station within a nature reserve in the Zambezi Valley were often infested with the immature stages of *A. tholloni* and that cases of heartwater occurred in these animals in the absence of any of the other known tick vectors.

### Dermacentor circumguttatus

Hoogstraal (1956) regarded *D. circumguttatus* as an elephant tick and reported that it was present on these animals in West and Central Africa, extending in the east only to the western regions of Uganda and Sudan. However, Yeoman and Walker (1967) reported it in elephants in the far north-western corner of Tanzania where it borders Uganda. Dias (1993) reported 10 males and two females of a subspecies that he referred to as *D. circumguttatus cunhasilvai* from elephants in the Mossurize region of Manica Province, Mozambique. *Dermacentor circumguttatus* has not been collected from this locality since then, nor were any of the elephants examined in the present study in the Reserva Nacional do Niassa, Mozambique, infested with it.

### *Rhipicephalus* species

We believe that the occurrence of *R. appendiculatus* on elephants represents an unusual infestation. *Rhipicephalus compositus*, of which a single collection was made, has been collected from thick-skinned animals, rhinoceroses, warthogs, bush pigs and buffaloes. While a large number of collections have also been made from cattle, the occurrence of this species on an elephant should be regarded as rare (Walker et al. 2000).

*Rhipicephalus humeralis* is an ornate *Rhipicephalus* species and is only present in mainland Tanzania, Kenya and Somalia (Walker et al. 2000). In Kenya, it occurs in the coastal region and bushed and wooded grassland in the south and central regions. Walker (1974) recorded 16 collections from cattle, 28 from elephants and nine from black rhinoceroses in Kenya, with one or two collections from other domestic and wild animals. *Rhipicephalus maculatus* is present in coastal woodland and bush and adjacent inland from north-eastern KwaZulu-Natal to southern Somalia (Walker et al. 2000). In addition to the coastal regions of Kenya, Tanzania and Mozambique, it is also present in bushed and wooded grassland in south and central Kenya as well as in woodland and bush adjacent to the coastal regions of Mozambique and Tanzania (Walker et al. 2000). It is a large ornate species with stout mouthparts and has been collected from hosts with thick hides: elephants, white and black rhinoceroses, bush pigs and buffaloes (Baker & Keep 1970; Horak et al. 1983, 2017; Horak, Boomker & Flamand 1991; Kariuki et al. 2012).

*Rhipicephalus praetextatus* is present in central and southern Ethiopia and northern Somalia (Walker et al. 2000). Ticks originally identified as *R. simus* and now presumed to be *R. praetextatus* have been recorded in southern Somalia, and practically throughout Kenya and Tanzania (Walker et al. 2000). Walker et al. (2000) reported 12 collections of *R. praetextatus* (species unconfirmed) from elephants in East Africa, and it has subsequently been collected from elephants in the Ngorongoro Crater, Tanzania (Fyumagwa et al. 2007). In the current study, *R. praetextatus* was collected from 11 elephants.

*Rhipicephalus pulchellus* is widespread in Eritrea, eastern Ethiopia, Somalia, east of the Great Rift Valley in Kenya and north-eastern Tanzania (Walker et al. 2000). Kariuki et al. (2012) collected *R. pulchellus* from cattle and sympatric buffaloes in south-eastern Kenya. This ornate tick species infests a multitude of domestic and wild animals among which Walker et al. (2000) reported three African elephants. Seven elephants in our study were infested with *R. pulchellus*. Although *Rhipicephalus dux* was not collected in the present survey, Walker et al. (2000) have recorded its presence on elephants. It is found in the Democratic Republic of Congo, particularly in the north-east, in Rwanda and in north-western Uganda (Matthysse & Colbo 1987). Thus, the four ornate *Rhipicephalus* spp. have all been collected from elephants.
